# Molecular Detection of *Enterocytozoon bieneusi* in Free-Range Sheep and Domestic Dogs from the Greater Hinggan Mountains Area of China

**DOI:** 10.3390/vetsci12090897

**Published:** 2025-09-15

**Authors:** Yanyan Jiang, Zhongying Yuan, Xu Wang, Hongling Zhang, Hao Zhou, Weiping Wu, Yujuan Shen, Jianping Cao

**Affiliations:** 1 National Key Laboratory of Intelligent Tracking and Forecasting for Infectious Diseases, NHC Key Laboratory of Parasite and Vector Biology, National Institute of Parasitic Diseases, Chinese Center for Disease Control and Prevention (Chinese Center for Tropical Diseases Research), World Health Organization Centre for Tropical Diseases, Shanghai 200025, China; jiangyy@nipd.chinacdc.cn (Y.J.); yuanzy@nipd.chinacdc.cn (Z.Y.); wangxu@nipd.chinacdc.cn (X.W.); zhouhao@nipd.chinacdc.cn (H.Z.); wuwp@nipd.chinacdc.cn (W.W.); shenyj@nipd.chinacdc.cn (Y.S.); 2 Jiagedaqi District Center for Disease Control and Prevention, Daxing’anling 165000, China; hljjgdqzhl@163.com

**Keywords:** *Enterocytozoon bieneusi*, sheep, dogs, genotype, ITS, zoonotic

## Abstract

*Enterocytozoon bieneusi* (*E. bieneusi*) is a globally distributed microsporidian that infects both humans and animals. However, reports on its occurrence in free-ranging sheep and domestic dogs from rural areas are limited. The objective of this study was to investigate the prevalence and genotype distribution of *E. bieneusi* among freely grazing sheep and household dogs in the Hinggan Mountain region of China. A total of 235 fecal samples were collected, consisting of 95 from sheep and 140 from dogs, and screened for *E. bieneusi* by nested PCR targeting the internal transcribed spacer region (ITS) within the nuclear ribosomal DNA (rDNA) gene. The total infection rate of *E. bieneusi* amounted to 3.0% (7/235), with sheep exhibiting a rate of 5.3% (5/95) and dogs demonstrating a rate of 1.4% (2/140). Sequence analysis identified two known genotypes in sheep (BEB6 and NESH4, both within phylogenetic group 2) and one known genotype in dogs (CHN-F1, phylogenetic group 1), with CHN-F1 being reported in dogs for the first time. Importantly, the detection of BEB6, a genotype previously associated with human infection, suggests that sheep could act as a potential reservoir contributing to zoonotic transmission and environmental contamination. These findings expand knowledge of the genotype spectrum of *E. bieneusi* in rural animal populations and provide evidence of possible cross-species transmission between humans and the surveyed animals in the region.

## Simple Summary

*Enterocytozoon bieneusi* (*E. bieneusi*) is a microsporidian parasite infecting humans and animals globally, yet data on its occurrence in free-range livestock and companion animals in rural areas remain limited. In this study, fecal samples from 95 free-range sheep and 140 dogs in two villages of the Greater Hinggan Mountains, China, were examined using nested PCR targeting the ITS region of the rDNA gene. The overall infection rate of *E. bieneusi* was 3.0% (7/235), including 5.3% (5/95) in sheep and 1.4% (2/140) in dogs. Sequence analysis identified two known genotypes in sheep (BEB6 and NESH4, phylogenetic group 2) and one in dogs (CHN-F1, phylogenetic group 1), with CHN-F1 reported in dogs for the first time. Notably, genotype BEB6 is a recognized zoonotic type, highlighting sheep as a potential source of human infection and environmental contamination. These findings expand current knowledge on the genetic diversity of *E. bieneusi* in rural settings and underscore the risk of cross-species transmission between humans and animals.

## 1. Introduction

Microsporidia are a diverse phylum of obligate intracellular parasitic fungi, comprising more than 1700 described species in over 220 genera, which infect a broad range of vertebrate and invertebrate hosts and are ubiquitous in the environment [1]. Among them, at least 17 species have been identified in humans, with four species—*Enterocytozoon bieneusi*, *Encephalitozoon cuniculi*, *Encephalitozoon intestinalis*, and *Encephalitozoon hellem*—being the most commonly reported [2,3]. Among them, *E. bieneusi* accounts for more than 90% of human microsporidiosis cases, with a pooled global prevalence of approximately 7.9% [4]. Immunocompromised or immunodeficient individuals, particularly HIV-positive patients and organ transplant recipients, are the most susceptible, with reported infection rates ranging from 2.5% to 76.9% [5,6]. In these populations, infection commonly results in persistent diarrhea, abdominal pain, wasting, weight loss, and malabsorption, whereas in immunocompetent individuals it usually manifests as self-limiting diarrhea or may even remain asymptomatic [7,8]. In addition to humans, *E. bieneusi* is found in more than 170 animals across 42 countries, highlighting its zoonotic potential [4]. Human infection typically occurs via the fecal–oral route, through ingestion of contaminated water or food, or by direct contact with infected individuals, including both humans and animals [4]. Spores, the infective stage of *E. bieneusi*, have also been detected in food, water, and various environmental samples [9], indicating multiple potential sources of exposure. Early evidence came from Peru, where Cama et al. (2007) identified an unusual genotype (Peru 16) in seven guinea pigs and a 2-year-old child from the same household, suggesting direct transmission across species [10]. Since then, increasing reports of overlapping genotypes in humans and animals have further supported the possibility of cross-species transmission [11,12,13]. Given that *E. bieneusi* can spread through direct human–animal contact [2], investigating its occurrence in hosts closely associated with humans is critical for clarifying infection sources and transmission pathways.

Accurate detection of *E. bieneusi* is hampered by the small size and unclear staining of its spores, rendering traditional microscopy insufficient. Consequently, molecular tools, particularly polymerase chain reaction (PCR) followed by sequencing of the internal transcribed spacer region (ITS) within the nuclear ribosomal DNA (rDNA) of *E. bieneusi*, serve as the gold-standard typing method [14]. To date, around 900 different genotypes of *E. bieneusi* have been discovered and categorized into 13 groups through phylogenetic analysis [15]. Notably, more than 94% of the genotypes identified belong to Group 1 and 2. The two groups encompass 93% of human pathogenic genotypes and 96% of zoonotic genotypes, earning them the designation of zoonotic groups [15]. By contrast, the remaining groups, ranging from Groups 3 to 13, typically include no more than ten genotypes that are predominantly isolated from specific hosts or wastewater and are thus designated as nonzoonotic groups [2,16]. Numerous hosts, particularly free-range animals, can propagate both zoonotic and nonzoonotic genotypes of *E. bieneusi* [15,16].

In rural areas, domestic animals such as sheep and dogs maintain close proximity with humans, potentially facilitating the transmission of zoonotic pathogens, including microsporidia [17]. To date, 69 genotypes of *E. bieneusi* have been identified in sheep, with 9 of these genotypes also identified in humans [15]. Notably, genotype BEB6, which has a broad distribution and the capacity to infect humans, highlights the significance of sheep in the dissemination of microsporidiosis. Similarly, dogs have been found to harbor at least 65 genotypes of *E. bieneusi* worldwide, with a substantial percentage being zoonotic and capable of infecting multiple hosts [15,18]. These observations underscore the crucial role of dogs in the transmission of *E. bieneusi* to humans and other animals. Understanding the epidemiology of *E. bieneusi* in these animals is pivotal for preventing its spread to humans and other animals.

In rural northern China, sheep farming serves as a primary source of income, and dogs are commonly kept in nearly every household as guardians of the home. Although *E. bieneusi* has been reported in a wide range of hosts worldwide, data on its occurrence and genetic diversity in free-range sheep and dogs in these regions remain scarce [19]. This knowledge gap limits our understanding of the epidemiology of *E. bieneusi* and its potential public health significance in areas where humans and domestic animals live in close contact. Based on this gap, we hypothesized that *E. bieneusi* is present in free-range sheep and dogs in northern China, and that these animals may harbor genotypes of zoonotic concern. To address this hypothesis, the present study aimed to investigate the infection rate and genotype distribution of *E. bieneusi* in free-range sheep and dogs from two villages in the Greater Hinggan Mountains region of China. Additionally, this study further assessed the zoonotic potential of the identified genotypes through sequence homology and phylogenetic analysis.

## 2. Materials and Methods

### 2.1. Fecal Sample Collection

Between 10 December 2018 and 31 October 2020, a total of 235 fecal samples were collected, including 95 from sheep and 140 from dogs, in two villages located in Jiagedaqi District of the Greater Hinggan Mountains region, China. This district consists of two townships (Jiabei Township and Baihua Township), comprising three villages and five villages, respectively. The two selected villages are representative of these townships. Together, the villages comprise approximately 120 households with a total population of approximately 500 residents. Due to the impact of African swine fever, no households in the investigated area keep pigs; instead, free-range sheep and household dogs are the main domestic animals. Ten households raise sheep, each maintaining 5–12 animals. During the daytime, sheep are allowed to graze freely in surrounding pastures, while at night, they are gathered within household fences. Additionally, approximately 100 families residing in the village own dogs, usually one or two per household. These dogs are commonly tethered near doorways to guard their property. All sampled animals appeared to be healthy. Fecal samples (~10 g each) were collected directly from the ground immediately after defecation and placed into sterile self-sealing bags. Samples were then transported to the laboratory within 2 days in ice boxes. The sample size was determined to cover all available sheep and dogs within the study villages. Information on age, sex, and detailed health status of the animals was not recorded, as the study was designed to focus on occurrence at the population level rather than individual-level risk factors.

### 2.2. DNA Extraction

Each fecal sample was homogenized with distilled water. The homogenates were then sieved and centrifuged at 1500× *g* for 5 min. From the processed material, approximately 200 mg of each homogenized sample was processed using the QIAamp DNA Mini Stool Kit (Qiagen, Germany), adhering strictly to the manufacturer’s instructions. To optimize the DNA yield, the lysis temperature was elevated to 95 °C. Subsequently, the DNA was eluted using AE elution buffer (200 mL) and stored at −20 °C until PCR analysis.

### 2.3. Detecting and Genotyping E. bieneusi

To detect and genotype *E. bieneusi*, nested PCR was performed using species-specific primers targeting an approximately 390 bp nucleotide fragment of the ITS region of the rDNA gene, which includes 76 bp of the 3′ end of the small subunit (SSU) rDNA gene, 243 bp of the ITS region, and 70 bp of the 5′ end of the large subunit (LSU) rDNA gene, as originally described by Buckholt et al. [20]. The primer sequences were as follows: external primers F1 (5′-GGTCATAGGGATGAAGAG-3′) and R1 (5′-TTCGAGTTCTTTCGCGCTC-3′), and internal primers F2 (5′-GCTCTGAATATCTATGGCT-3′) and R2 (5′-ATCGCCGACGGATCCAAGTG-3′) [20]. The PCR conditions for the two rounds were as follows: 35 cycles at 94 °C for 30 s, 57 °C for 30 s, and 72 °C for 40 s and 30 cycles at 94 °C for 30 s, 55 °C for 30 s, and 72 °C for 40 s, with both of them having an initial denaturation step at 94 °C for 5 min and a final extension step at 72 °C for 7 min [20]. For all PCR amplifications, TaKaRa TaqDNA Polymerase (Takara Biomedical Technology (Beijing) Co., Ltd., Beijing, China) was utilized. Both a negative control without DNA and a positive control with Peru6 genotype DNA from deer were included. The PCR products were then analyzed via 1.5% agarose gel electrophoresis and visualized via DNAGREEN staining (Tiandz, Inc., Beijing, China).

### 2.4. Sequencing Analysis

The PCR products exhibiting a positive reaction for *E. bieneusi* were subjected to direct sequencing at Sangon Biotech Co., Ltd., Shanghai, China, which is located in Shanghai, China. This sequencing was performed utilizing the Big Dye1 Terminator v3.1 cycle sequencing kit, which was produced by Applied Biosystems (Carlsbad, CA, USA). The raw sequences obtained from sequencing were aligned using Clustal X (1.83) software (available at http://www.clustal.org/) (accessed on 15 September 2024). Homology comparisons of *E. bieneusi* ITS were performed against the GenBank database via the Basic Local Alignment Search Tool (BLAST) (available at http://www.ncbi.nlm.nih.gov/BLAST/) (accessed on 15 September 2024). All the genotypes were identified only based on 243 bp of the ITS region of the rDNA gene of *E. bieneusi*, according to the established nomenclature system [21]. When ITS sequences were identical to previously reported genotypes, they were assigned the corresponding published names. In cases where a known genotype was detected in a novel host, the sequence was submitted to the GenBank database under the accession number PX136961.

### 2.5. Phylogenetic Analysis

To confirm the phylogenetic group designation and to assess the relationship between the identified genotypes and those already known, a phylogenetic tree was constructed using the neighbor-joining (NJ) method with the Kimura 2-parameter model in MEGA 7. Bootstrap analysis with 1000 replicates was used to determine support for the resulting tree.

### 2.6. Statistical Analysis

The 95% confidence intervals (CIs) were calculated using the OpenEpi software program (http://www.openepi.com/Proportion/Proportion.htm) (accessed on 20 August 2025).

## 3. Results and Discussion

This is the first report documenting the occurrence of *E. bieneusi* infection in sheep and dogs within the region. Analysis of 235 samples revealed that only 3.0% (7/235; 95% confidence interval: 1.3–5.8%) were positive for *E. bieneusi*, with sheep exhibiting a detection rate of 5.3% (5/95; 95% CI: 1.9–11.3%) and dogs showing a rate of 1.4% (2/140; 95% CI: 0.2–4.6%) (Table 1). A global pooled prevalence rate for *E. bieneusi* in sheep has been estimated to stand at 17.4%, while in China, the figure is slightly higher at 17.9% [17]. A comparative analysis across various regions of China has shown that the prevalence rate observed in the present study is notably lower than that reported in other provinces. For instance, Heilongjiang Province recorded rates varying between 4.4% and 51.7% [22,23], while Inner Mongolia exhibited a particularly high prevalence of 69.3% [24]. Tibet’s rates fell within the range of 6.4% to 23.4% [25,26], Ningxia reported rates ranging from 12.2% to 47.1% [23,27], Jiangsu had a prevalence of 36.5% [28], Shanxi showed a rate of 34.2% [29], Yunnan reported a prevalence of 12.3% [30], and Shandong had a lower rate of 1.6% [31]. Moreover, besides China, reports of *E. bieneusi* infection in sheep are limited to only a few countries, including Brazil (19.2%) [32], Egypt (6.7%) [33], Iran (10.0%) [34], and Sweden (45.0%) [35], all of which have documented markedly higher prevalence rates compared with our results. These variations in infection rates could be attributed to several factors, including climatic variations, animal husbandry practices, detection methods, and the number of animals examined. Here, all the positive samples originated from Xingfu village geographically. Indeed, prior studies have also demonstrated significant variations in infection rates even among different studies conducted within the same region, such as two studies from Heilongjiang Province of China reporting distinct infection rates of 4.4% (2/45) and 22.5% (31/138) [22,36]. Therefore, increasing the number of survey samples and expanding the geographical scope can contribute to understanding the actual prevalence of *E. bieneusi* in specific animal hosts within a region, which is crucial for preventing and controlling its spread.

In the present study, the infection rate of dogs infected with *E. bieneusi* was relatively low, at only 1.4% (Table 1). Previous research has demonstrated that the infection rate varies significantly across countries or regions, ranging from 0.8% in Spain [37] to 39.6% in China [38]. However, disparities in infection rates have been reported in different studies within the same country. For instance, while one study in Spain reported a low infection rate of 0.8% [37], three other studies conducted in the same country reported infection rates of 19.2% [39], 11.8% [40], and 8.7% [41]. Similarly, only one study reported the prevalence of *E. bieneusi* in dogs in countries such as Korea (43.8%) [42], Colombia (15.0%) [43], Poland (4.9%) [44], Switzerland (8.3%) [45], Australia (4.4%) [46], and Egypt (13.0%) [33]. Therefore, these figures cannot be considered representative of the overall infection situation in those regions. In China, at least 14 provinces have reported cases of infected dogs, with infection rates ranging from 2.5% in Heilongjiang [47] to 40.7% in Hebei [48]. Overall, the infection rate observed in the present study was lower than that previously reported in China, which could be attributed to the source and mobility range of the dogs sampled in this study. Here, the collected dogs were scattered among different families and had no more than two dogs per family. These dogs have a small, consistent activity area and limited contact with the external environment, potentially reducing the risk of infection. However, it is noteworthy that this study still revealed the presence of *E. bieneusi* infection in dogs in the investigated areas. Given that people inevitably come into contact with dogs, it is particularly important to properly manage dog feces to prevent environmental contamination and infection of other animals or humans. This point cannot be ignored.

Through sequence analysis of seven *E. bieneusi* isolates obtained in this study, three genotypes were identified: CHN-F1, NESH4, and BEB6 (Table 1, Figure S1). Among the samples tested, genotype BEB6 was identified in four sheep, genotype NESH4 was present in one sheep, and genotype CHN-F1 was detected in two dogs. Consistent with previous epidemiological surveys, genotype BEB6 is globally the most frequently reported genotype among sheep, and our findings further confirm its prevalence in sheep herds [15]. This genotype is not confined to sheep but also dominates in other ruminant species, such as goats, cattle, camels, donkeys, horses and deer [49]. Notably, genotype BEB6 poses a significant zoonotic threat due to its ability to infect children residing in the Shanghai and Henan regions of China [50,51]. Furthermore, it is widely distributed among approximately 15 different animal species from five distinct countries, including foxes, cats, dogs, chinchillas, whooper swans, pet birds, flies, golden takins, nonhuman primates, and captive wild animals in zoos [15,49]. Additionally, it is present in the environment, such as in raw wastewater and on the surfaces of fruits and vegetables as well as in the raw milk of cattle, sheep, and water buffaloes [52,53]. Therefore, sheep infected with this genotype could have significant public health implications. Additionally, the NESH4 genotype, which was also obtained from sheep in this study, was previously reported in sheep from Heilongjiang Province, China [54]. To date, this genotype has not been reported to infect humans or other hosts, and further studies are needed to confirm its potential host specificity.

In our study, two dog kennels harbored the same genotype, CHN-F1, which was initially identified in a farmed fox-derived isolate of *E. bieneusi* in Heilongjiang Province, China [55]. Subsequently, it has been sporadically detected in foxes in Xinxiang and has become dominant in infected raccoon dogs in northern China [56,57]. These findings suggest that this genotype may be specific to canine animals. However, recently, CHN-F1 has also been reported in feral and captive pigeons in Central Europe, indicating a potentially broader host range [58]. Importantly, our study represents the first detection of CHN-F1 in dogs, thereby further expanding the known host range of this genotype. Additional studies are needed to clarify whether CHN-F1 has the potential to infect humans.

## 4. Conclusions

The present study represents the first report of *E. bieneusi* infection in free-ranging sheep and domestic dogs in the Greater Hinggan Mountains. Three genotypes were identified, including BEB6, CHN-F1, and NESH4. Genotype BEB6 identified here has previously been detected in humans and some animals in other countries, which suggests that sheep may act as reservoirs for this genotype and facilitate its transmission to humans. Notably, CHN-F1 was identified in dogs for the first time in this study, thereby expanding the known host range of this genotype. By contrast, the zoonotic potential of CHN-F1 and NESH4 remains unclear. Although the overall infection rate of *E. bieneusi* in this study was relatively low, the detection of zoonotic genotypes and the clustering of all genotypes within phylogenetic Groups 1 or 2 suggest that sheep and dogs in this region may serve as potential reservoirs for human infection. These findings highlight the importance of strengthening fecal management and improving hygienic practices to mitigate the risk of zoonotic transmission.

## Figures and Tables

**Table 1:** Detection rates and genotypes of *E. bieneusi* in goats and dogs from two villages in the Greater Hinggan Mountains of China.

Host	Xingfu Village	Baihua Village	Total	
	Detection Rate (n)	Detection Rate (n)	Detection Rate (n)	Phylogenetic Group: Genotype (n)
Sheep	8.1% (5/62)	0/33	5.3% (5/95)	2: NESH4 (1), BEB6 (4)
Dog	2.5% (2/80)	0/60	1.4% (2/140)	1: CHN-F1 (2)
Total	3.0% (7/235)	0/93	3.0% (7/235)	1: CHN-F1 (2); 2: BEB6 (4), NESH4 (1)

## Data Availability

The raw data supporting the conclusions of this article will be made available by the authors on request.

## Supplementary Materials

The following supporting information can be downloaded at https://www.mdpi.com/article/10.3390/vetsci12090897/s1: Figure S1: Phylogenetic relationships of *Enterocytozoon bieneusi* genotypes identified in this study with known genotypes deposited in GenBank, as inferred by a neighbor-joining analysis of the ITS rDNA gene sequences based on genetic distances calculated with the Kimura-2-parameter model. Bootstrap values (%) from 1000 replicates are shown at the nodes. Each sequence is labeled with its GenBank accession number, genotype designation, and host origin. The genotype CSK2 (KY706128) from white kangaroo was used as the outgroup. Black circles preceding genotype names indicate known genotypes detected in a novel host in the present study.
