# Supplementary material for: Molecular Detection of Enterocytozoon bieneusi in Free-Range Sheep and Domestic Dogs from the Greater Hinggan Mountains Area of China
Source: Vet Sci. 2025 Sep 15;12(9):897. doi: 10.3390/vetsci12090897 (PMC12474382; doi:10.3390/vetsci12090897)
Supplement: Supplementary file 1 [file vetsci-12-00897-s001.zip › vetsci-3790175-supplementary.pdf]

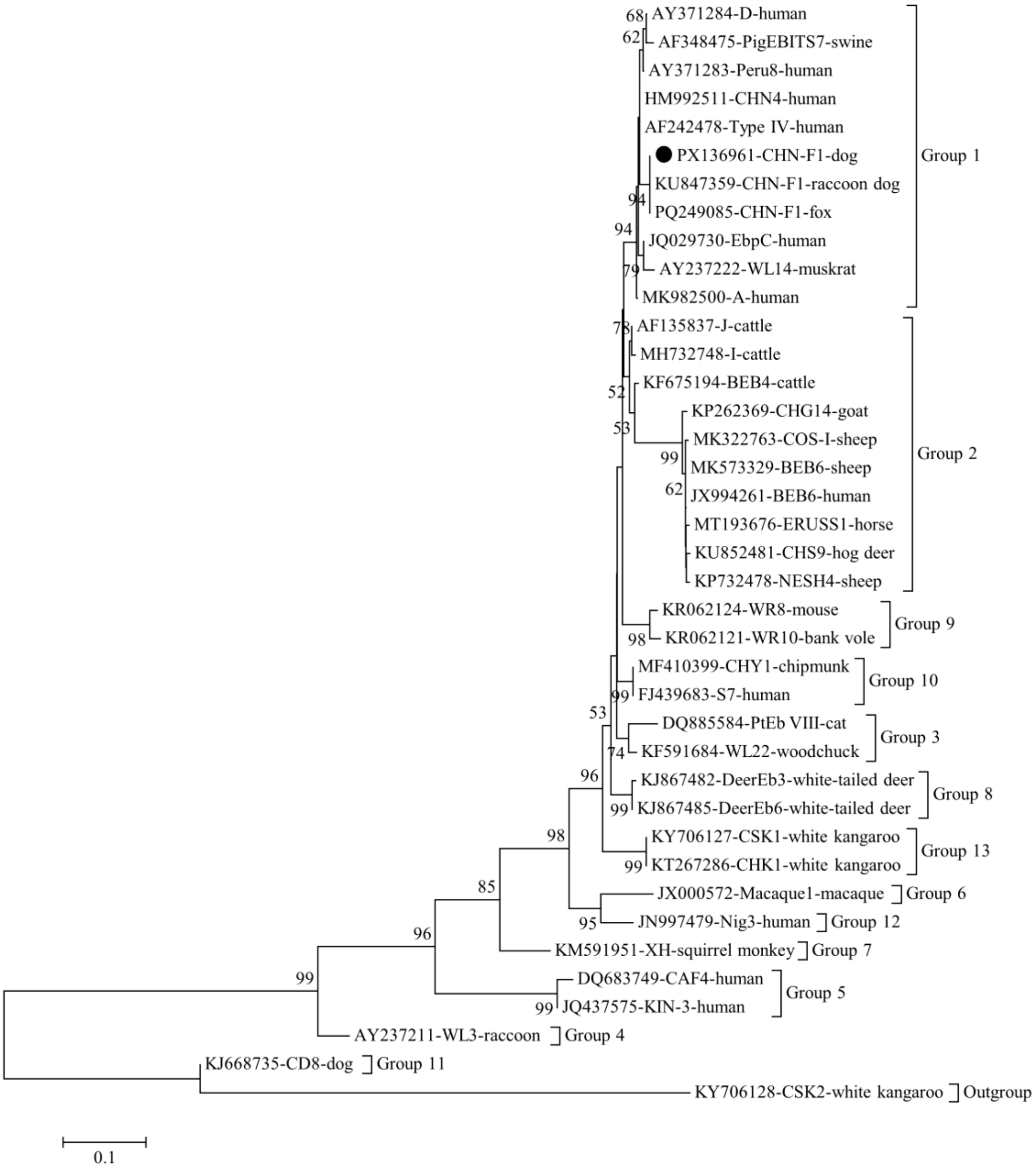

Figure S1: Phylogenetic relationships of *Enterocytozoon bieneusi* genotypes identified in this study with known genotypes deposited in GenBank, as inferred by a neighbor-joining analysis of the ITS rDNA gene sequences based on genetic distances calculated with the Kimura-2-parameter model. Bootstrap values (%) from 1,000 replicates are shown at the nodes. Each sequence is labeled with its GenBank accession number, genotype designation, and host origin. The genotype CSK2 (KY706128) from white kangaroo was used as the outgroup. Black circles preceding genotype names indicate known genotypes detected in a novel host in the present study.
